# Cancer Therapy by Silver Nanoparticles: Fiction or Reality?

**DOI:** 10.3390/ijms23020839

**Published:** 2022-01-13

**Authors:** Dávid Kovács, Nóra Igaz, Mohana K. Gopisetty, Mónika Kiricsi

**Affiliations:** 1 Department of Biochemistry and Molecular Biology, University of Szeged, Közép Fasor 52, H-6726 Szeged, Hungary; kvcs.david@gmail.com (D.K.); noraigaz@gmail.com (N.I.); jaisairam205@gmail.com (M.K.G.); 2 CNRS, Institut de Pharmacologie Moléculaire et Cellulaire, Université Côte d’Azur, 660 Route des Lucioles, 06560 Valbonne, France; 3 Interdisciplinary Center of Excellence, Department of Applied and Environmental Chemistry, University of Szeged, Rerrich Béla Tér 1, H-6720 Szeged, Hungary

**Keywords:** silver nanoparticles, metal nanoparticles, cancer therapy

## Abstract

As an emerging new class, metal nanoparticles and especially silver nanoparticles hold great potential in the field of cancer biology. Due to cancer-specific targeting, the consequently attenuated side-effects and the massive anti-cancer features render nanoparticle therapeutics desirable platforms for clinically relevant drug development. In this review, we highlight those characteristics of silver nanoparticle-based therapeutic concepts that are unique, exploitable, and achievable, as well as those that represent the critical hurdle in their advancement to clinical utilization. The collection of findings presented here will describe the features that distinguish silver nanoparticles from other anti-cancer agents and display the realistic opportunities and implications in oncotherapeutic innovations to find out whether cancer therapy by silver nanoparticles is fiction or reality.

## 1. Introduction

In the past decade, significant efforts have concentrated on fundamental and translational research to introduce nano-sized materials to cancer medicine [1]. As a result, to date, several nanomaterial-based treatment modalities have been translated into clinical trials to overcome cancer [1]. Although these are mostly liposome-encapsulated chemotherapy drugs, recently, other nanomaterials—metal-based nanostructures, in particular—have emerged as therapeutically useful agents. Among them, due to their well-recognized anti-microbial activities silver nanoparticles (AgNPs) are the most widely used in various clinical applications [2]. In addition to anti-microbial activities, AgNPs possess unique cytotoxic features against mammalian cells as well, which properties render silver-based nanoparticles potentially applicable in tumor therapy. While a rapidly growing number of scientific data support their possible application as anti-cancer agents, to develop silver nanoparticle-based therapeutic modalities with high efficacy and reliable safety, numerous issues should be elucidated in advance.

The therapeutic potential of nanoparticulated silver relies on its unique mode to induce cell death in mammalian cells. Regardless of their physical and chemical properties, such as heterogeneity in size, shape, and capping material, their way of action to induce cancer cell death is rather dogmatic. Following their uptake, mostly by endocytosis-related mechanisms, AgNPs are collected in endosomes, of which the organelles are then directed to lysosomal fusion. The lysosomal acidic environment leads to an increased release of silver ions from the AgNPs, of which the reactive ions then unbalance cellular homeostasis and—based on the biological feature of the targeted cell—which leads to apoptotic cell death [3]. This type of action is traditionally referred to as the “Trojan-horse”-type mechanism and implies that the cytotoxic feature of AgNPs emerges only following their uptake by the cells [4,5].

**Figure 1:**
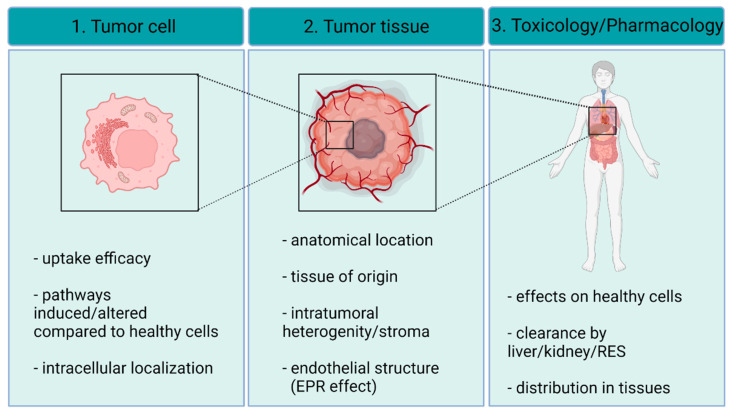
Before their clinical application, detailed understanding of the AgNP-triggered effects on the level of single cells, cancer tissues and organs is mandatory. In this review, we discuss the latest knowledge accumulated on AgNP–cancer interactions at the abovementioned three organization levels. The figure was created with BioRender.com.

To exploit the “nano” nature of the materials, nanoparticles are generally applied to deliver cytotoxic drugs to targeted cells. AgNPs also own this beneficial “nano” property, and all the advantages of a nano-system, e.g., the size-surface ratio or tunable surface, as well as intrinsic cytotoxic features, through reactive Ag ions (“silver” property). Therefore, AgNPs can be considered a two-in-one therapeutic system. Despite these favorable features, AgNPs appear to be somewhat toxic to healthy tissues [6,7]; therefore, it is important to assure that the applied nanoparticles accumulate preferentially only or mainly in the cancerous tissue, leaving other non-target organs unaffected. To achieve this, multiple passive and active-targeting methods need to be, and in fact, have already been applied for silver-based nanosystems. Tumor tissues contain heterogeneous cell populations and the interactions between non-cancerous and cancer cells have a crucial impact on the evolution of the disease as well as on the therapy outcome [8]. Therefore, to develop novel treatment regimens employing such nanoparticles, it is essential to understand how AgNPs influence the crosstalk between cancer cells and the elements of the tumor stroma once AgNPs reach the tumor microenvironment. Finally, the biological nature of the tumor cell certainly dictates the efficiency of AgNPs; thus, mechanistic understanding of how cancer cells with different genetic profiles respond to AgNP exposures is in fact mandatory. In this review, we summarize our current view on the implication of AgNPs in cancer therapy and we discuss some future research directions that might help to develop novel efficient tools for rational cancer therapy (Figure 1).

## 2. Synthesis and Characterization of AgNPs

Depending on the biological purpose, AgNPs with specific properties can be designed and produced with appropriately controlled synthesis methods. In the last decade, a plethora of techniques and synthesis procedures were introduced that are now available for the preparation of metal nanoparticles with adequate size, shape, surface charge and functionalization. The most generally utilized synthesis methods are mostly physical, chemical and biological approaches [9]. Physical synthesis usually represents a top-down method, where nanosized particles are obtained from the bulk material for example with evaporation-condensation or laser ablation [10,11]. The most important advantage of these production regimens is that high amounts of the product can be prepared without chemical contamination; however, the stabilization of the nanoparticles is often not satisfyingly assured [9]. The chemical and biological synthesis methods of nanomaterials utilize a bottom-up approach. The majority of nanoparticles produced for industrial or scientific purposes is prepared by chemical reduction. In this case, metal salts are dissolved in proper aqueous or organic solvents and the metal ions are reduced chemically in the presence of reducing and capping agents to yield a stable colloidal solution. However, the final colloid may contain toxic by-products which obviously limit the biological applicability of the nanomaterials [9]. Besides the classical synthesis methods, a growing number of studies highlight the beneficial application of biological synthesis approaches using “green” materials during the preparation of nanomaterials, thereby avoiding toxic by-products in the resulting colloid. Green synthesis procedures mostly utilize plant extracts originating from different plant parts, such as leaf, seed or root. Moreover, green nanoparticle synthesis can be achieved via microorganisms as well, mainly by bacteria or fungi, although such methods need further considerations if the nanoparticles are aimed for human therapeutic purposes [12]. For more details on green metal nanoparticle synthesis, the reader is referred to Rónavári et al. [12].

Following AgNP synthesis—independently from the type of the production method—a detailed characterization is obligatory to ensure the biological applicability of the nanomaterials. The physico-chemical properties of the nanoparticles, such as size, shape, surface charge and grade of dispersity, can inherently dictate their biological effects; thus, such a description should be the first step in nanoparticle characterization. The most commonly used techniques are transmission and scanning electron microscopy, UV-Visible spectroscopy (UV-Vis), dynamic light scattering (DLS) and zeta potential measurements (ZP), which give appropriate information about the size, morphology, stability, mono- or polydispersity and surface properties of the particles. Moreover, more precise qualitative analysis can be achieved by Fourier transformed infrared spectroscopy (FT-IR), inductively coupled plasma mass spectrometry (ICP-MS), Raman spectroscopy and X-ray photoelectron spectroscopy (XPS) to obtain information about the chemical composition, surface residues and functional groups on the nanoparticle surface [12]. In Table 1, the most frequently utilized characterization methods are summarized.

## 3. AgNP–Cancer Interactions

### 3.1. Targeting AgNPs to the Tumor Tissue

**Figure 2:**
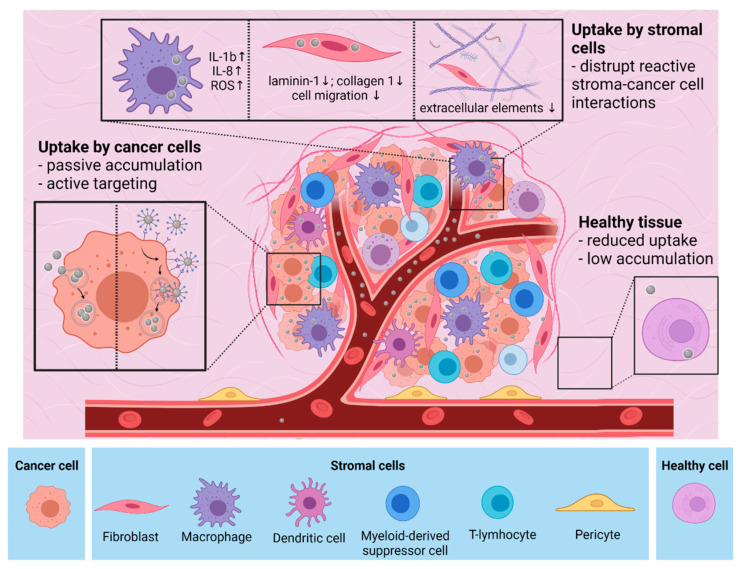
Interactions of AgNPs with cancer and stromal cells of the tumor tissue and with healthy cells. AgNPs are accumulated in tumor tissue passively or can be targeted to the tumor actively. AgNPs affect the stroma-cancer cell communication. Reduced accumulation of nanoparticles is observed in healthy tissues. The figure was created with BioRender.com.

Several research groups demonstrated the exceptional potential of AgNPs as anti-cancer agents; however, they have also emphasized that the toxic behavior of these nanomaterials renders them somewhat similar to conventional chemotherapy drugs. Nevertheless, the main difference between AgNPs and conventional small molecular drugs lies within the “nano” nature of AgNPs, which ultimately helps to reduce the severity of undesired side effects. As a result of this nano-feature, AgNPs can be targeted either passively or actively to the tumor tissues, where they can, thus, accumulate in high concentrations (Figure 2). Passive accumulation is based on the unique architecture of the tumor tissue, where neo-angiogenesis leads to an atypical endothelial layer and to fenestrated vasculature, which together with the impaired lymphatic drainage guides the penetration and accumulation of nano-sized materials within the cancerous tissues [26]. This phenomenon is called the enhanced permeability and retention (EPR) effect, which has been exploited for drug design in the nanomedicine field [27]. It has also been proposed that shape, size, and capping materials applied on the surface of the nanoparticles can help to optimize their passive accumulation. We have to note, however, that these observations have been established mostly using xenograft models, where the tissue structure is known to be different from that of naturally developing human tumors [28]. For instance, it has been found that nanomedicine accumulation in the tumors is more efficient in small animal xenograft models than in naturally occurring tumors in patients [29,30]. However, evidence suggests that EPR effect drives drug accumulation in tumors growing in patients as well, but its efficacy heavily varies on the specific anatomical features of the given tumor [31]. Therefore, while the EPR effect may act as a decisive concept upon nanoparticle tumor-targeting, it must be regarded cautiously as it has become a focus of debate recently. 

As an alternative to passive targeting, active targeting strategies have been proposed to be viable approaches to increase the cancer specificity of silver nanoparticles. Upon active targeting, a cancer cell-specific moiety is bound onto the nanoparticle surface to facilitate the uptake of the nanomaterials by the desired population of cells (reviewed by Yoo, Jihye et al. [32]). Cancer-specific receptor ligands, as well as antibodies, can be typically employed; however, successful application of cell-penetrating peptides on AgNP surfaces has also been reported to enhance the therapeutic efficacy of such nanomaterials [33]. An altered surface glycosylation pattern is a hallmark of cancer cells, particularly of breast cancer, where it is used frequently as a prognostic marker [34]. Soybean agglutinin has a specific binding toward such cancer cell surface patterns, thus providing an exploitable targeting strategy for this group of malignant cells. In line with this, it was found that soybean agglutinin-conjugated silver nanoparticles were specifically cytotoxic to breast cancer cell lines (MDA-MB-231 and MCF-7), but not to non-cancerous breast epithelial cells (MCF-10A) [35]. To achieve an elevated uptake efficiency, folic acid-conjugated AgNPs have likewise been generated and tested on folate receptor-overexpressing cancer cells [36,37]. It has been reported that via amide bonds, AgNPs can be functionalized with folic acid, thereby increasing their specificity toward folic acid receptor overexpressing tumors [38]. In another study, AgNPs were combined with graphene-oxide and the chemotherapy drug methotrexate was used as a targeting ligand since it interacts with folic acid receptors, which are usually overexpressed in cancer cells [39]. Importantly, folate receptors are typically internalized via caveolin-dependent endocytosis [40]; however, this pathway often bypasses lysosomes [41]. The acidic environment of lysosomes is a prerequisite for Ag ion release; nevertheless, in these studies, folic acid-targeted AgNPs were reported to be more cytotoxic than their non-targeted counterparts. Hence, further studies should be conducted to clarify whether folic acid-dependent AgNP uptake still leads to lysosomal entrapment or folic acid-conjugated AgNPs are able to release Ag ions in a lysosome-independent way as well.

Although active targeting strategies are promising and represent a dynamically growing field of nanomedicine, they have numerous limitations, and several aspects should be taken into consideration upon the selection of the active targeting moiety. One of these is the biological nature of the treated tumor itself. For instance, to ensure the selection of the most specific targeting strategy, a complete receptor profiling of the malignant tissue would be beneficial despite being financially demanding. As an alternative approach, multiple targeting ligands could be applied on the nanoparticle surface representing a collection of the most frequently detected cancer-receptor ligands. 

Another concept to contemplate in therapeutic nanomaterial design is the formation of a biomolecular corona. A protein corona, or more specifically a biomolecular corona, is formed on the surface of the nanoparticles whenever they enter biorelevant media, such as blood. The biomolecular corona affects not only the physical properties of the nanoparticles but significantly influences their interactions with cells, and thus, the targeting of nanoparticles to cancer cells [42,43,44]. In blood, typically, serum proteins and smaller organic compounds, such as glucose, amino acids, and lipids, are adsorbed to the AgNP surface, forming a dynamic biomolecular layer around the nanoparticles, and covering the nano surface [45]. This feature is highly advantageous because such a biomolecular corona not only suppresses particle aggregation, but also facilitates the cellular internalization of nanoparticles [46]. However, it should be noted that the biomolecular corona acts as a double-edged sword, since the extensive layer of proteins and other biomolecules might cover and bury the targeting groups bound to the nanoparticle surface, rendering them entirely ineffective [47]. Nevertheless, size/shape features and surface charges may influence the composition and the thickness of the biomolecular corona, allowing the possibility for optimization. Additionally, the biomolecular corona could be bypassed via the application of long adaptor molecules forming bridges between the nanoparticle surface and the targeting ligands. With such linkers, the active targeting ligands might overhang the corona; thus, they can be recognized by cancer cell receptors.

Oftentimes, AgNPs themselves are delivered within complex nanosystems. In such cases, AgNPs represent the active component of the nanocomplexes, and their targeted delivery is ensured by the application of other carrier platforms. For example, silver nanoparticles have been embedded into mesoporous silica, which were functionalized with transferrin. Using this nanocomplex, only transferrin-receptor overexpressing cancer cells were able to take up the nanocomposite, yielding a specific delivery of cytotoxic AgNPs [48]. In another study, AgNPs were incorporated into polymeric nanoparticle carriers, which were functionalized with a 36 amino acid-long peptide, chlorotoxin. Chlorotoxin was identified as the venom of the scorpion *Leiurus quinquestriatus* and it interacts specifically and selectively with a matrix-remodeling enzyme MMP2, which is often presented on the surface of several cancer cells such as glioblastoma cells. With this indirect active targeting method, AgNPs were co-delivered with the drug alisertib, and the nanocomplex successfully reduced in vivo glioblastoma growth [49].

### 3.2. Interaction of AgNPs with the Tumor Stroma

Once AgNPs reach the tumor microenvironment, they inevitably interact with the cellular and non-cellular components of the tumor stroma (Figure 2). Since the interaction between stromal components and cancer cells inherently drives the evolution of the cancerous tissue, therefore, upon drug development, the effects of the novel therapeutic agents have to be carefully evaluated on the tumor microenvironment as well. However, to date, only a very limited number of studies deal with the impact of silver nanoparticles on the tumor stroma. Recently, we found that silver-coated gold nanoparticles can disrupt the interaction between cancer-associated fibroblasts and carcinoma cells, leading to a suppressed metastatic activity in vivo [50]. It has also been demonstrated that fibroblasts treated with AgNPs decreased laminin-1 and collagen-1 production and cell migration, further demonstrating the potent inhibiting effects of AgNPs on stromal fibroblasts [51]. Although these results are promising, additional mechanistic studies should be performed on various stromal cell types, such as macrophages or endothelial cells, in order to estimate more properly the impact of AgNPs on the cellular niche of the tumor. Importantly, it has been shown that AgNPs have a more potent in vivo anti-tumor effect in immunocompetent mice than in their immunodeficient counterparts, suggesting that AgNP administrations activate the anti-tumor immunity of the tumor microenvironment [52]. In fact, AgNP-triggered effects in tumor-associated macrophages have not been published so far, although it was shown that AgNPs of various sizes increase IL-1b and IL-8 mRNA levels and induce reactive oxygen species (ROS) production in macrophages [53]. This is extremely relevant from a therapeutic point of view since the above-described features are characteristics of macrophages of M1 polarization, cells that are capable of initiating anti-tumoral responses [54]. 

Non-cellular elements of the tumor stroma may also influence tissue and cell penetration as well as certain intracellular events triggered by nanoparticles. Stiffness of the extracellular matrix dictates epithelial-to-mesenchymal transition and metastasis and might also affect intratumoral nanoparticle distribution. Importantly, AgNPs are shown to suppress the secretion of extracellular elements; thus, they can potentially influence the activity of matrix-producing cells within the tumor microenvironment [55]. Furthermore, it has been shown that 20 nm AgNPs increase the MMP-9 secretion and, consequently, the gelatinase activity of polymorphonuclear neutrophil cells, implicating that AgNPs in the tumor microenvironment might efficiently modify the composition of the extracellular matrix [56] (Figure 2).

AgNPs can be designed to exert their effect only when they reach the tumor microenvironment. The internal niche of the tumors is slightly acidic compared to that of normal tissues [57], and this difference in pH can affect the nanoparticle–stroma interaction. Nevertheless, this difference in acidity can be harnessed for the selective release of drugs from the AgNP surface [58]. In a recent study, AgNPs were synthesized from an aqueous mixture of silver and alendronate under microwave irradiation and were further conjugated to the chemotherapeutic agent doxorubicin [59]. Later, these nanoparticles exhibited a pH-triggered release of doxorubicin in HeLa cells that potentiated the cytotoxicity of doxorubicin.

### 3.3. Uptake of AgNPs by Cancer Cells

Since the toxic effect of AgNPs on the exposed cells is primarily mediated by the internalized nanoparticles, it is critical to understand the mechanisms which regulate the uptake of AgNPs by cancer cells. It has been reported that the toxicity of AgNPs on various cell lines depends rather on the uptake efficiency of the intact nanoparticles than on the individual sensitivity of a given cancer cell to Ag ions, which features also highlight the inherent detrimental effect of intracellularly released Ag ions [60]. Generally, AgNPs are taken up by endocytotic mechanisms, and the fact that electron microscopic imaging typically shows AgNPs localized in endocytic vesicles greatly supports this view [61]. However, not all cancer cell types endocytose nanomaterials with the same efficiency. For example, it has been shown that glucose and lactose-capped AgNPs are taken up similarly by L929 fibroblast cells, while A549 carcinoma cells endocytose lactose-modified AgNPs with higher efficiency than their glucose-modified counterparts [62]. Furthermore, to maintain elevated growth stimuli, endocytotic pathways are impaired in some cancer cells; thus, in such cases, alternative uptake mechanisms should mediate AgNP uptake [63].

Besides the different endocytic capacities of the various cancer cells, the physicochemical parameters of the applied AgNPs also dictate their cellular entry. The chemical characteristics of the capping materials on the outer layer of the nanoparticle shape the biomolecular corona assembly and, thereby, the recognition of AgNPs by the cancer cells. For example, it has been shown that higher amounts of tannic acid-coated AgNPs were taken up by A549 cells than citrate- or PVP-coated nanoparticles [64]. Nevertheless, when the uptake of PVP- and citrate-coated AgNPs were compared, no evident difference in the internalization efficiency or intracellular localization was observed, indicating that apart from capping materials, other factors may also influence the AgNP–target cell interactions [65]. Notably, both citrate and PVP are providing a negative surface charge to AgNPs, highlighting that not primarily the chemical nature but rather the net charge of the AgNPs dictates their penetration efficiency.

Nanoparticle size has also been recognized as an important factor determining cellular uptake. A total of 5, 20, 50, and 100 nm AgNPs were added to methyl-beta-cyclodextrin- (caveolin-mediated endocytosis inhibitor)- and 5-(N-ethyl-N-isopropyl)-Amiloride (macro- and pinocytosis inhibitor)-treated cells, and it has been found that the uptake of smaller nanoparticles was suppressed by the caveolin-mediated endocytosis inhibitor treatments, while the macro- and pinocytosis inhibitors decreased predominantly the uptake of larger AgNPs, 50 and 100 nm [66]. We found that 5 nm and 35 nm AgNPs trigger identical apoptotic pathways in osteosarcoma cells, showing that once the smaller or larger AgNPs are internalized, their size does not affect the way of action [67]. P. Orlowski et al. also demonstrated that heterogeneous uptake mechanisms may be involved in AgNP uptake [68]. They tested several endocytosis inhibitors on the uptake efficacy of tannic acid-modified AgNPs by dendritic cells and they found that of all the applied inhibitors, only MDC—a clathrin-mediated endocytosis inhibitor—and CChD—a macropinocytosis and phagocytosis inhibitor— significantly suppressed the uptake of the nanoparticles. Phagocytosis is a specific uptake mechanism classically considered as a process specific to immune cells, typically by macrophages, dendritic cells, and B lymphocytes to trap and filter out pathogens, damaged cells, and exogenous materials, such as pollens. AgNPs are reported to be taken up intensively via phagocytosis by macrophages [69]. This feature is mostly disadvantageous, since AgNPs can be filtered out from the circulation via the phagocytotic cells of the reticuloendothelial system (RES) before they could reach the tumor. To overcome this, AgNPs have been successfully PEGylated, since the presence of PEG on the nanoparticle surface can inhibit opsonization and, thus, immune clearance [70,71]. Importantly, Guo et al. showed that PEGylated AgNPs can be internalized and concentrated to endosomes by K562 chronic myeloid leukemia cells incapable of phagocytosis, further supporting the notion that cancer cells can utilize various pathways to take up AgNPs [72].

Cell-penetrating peptides (CPPs) are small cationic peptides generally rich in arginine and lysine, which are reported to be able to translocate themselves and their associated moieties through the plasma membrane of cells [73]. This translocation is achieved by various mechanisms, such as endocytosis induction or direct penetration. CPPs have been widely used to assist the intracellular transport of different types of nanoparticles [73,74]. Farkhani et al. reported that AgNPs bioconjugated with CPPs greatly improved the internalization and cytotoxicity of AgNPs. They used thiol-functionalized AgNPs, capped with 3-mercaptopropionic acid (MPA). These MPA-AgNPs were bioconjugated to CPPs through EDC/NHS method and then their cytotoxicity was tested on MCF-7 breast adenocarcinoma cells [75]. Liu et al. have tested AgNPs of 8 nm in size modified with TAT—a frequently used cell penetrating peptide—to attenuate multidrug resistance in cancer cells. Through a stable Ag-S bond, the thiolated peptide was stably conjugated to the AgNP surface and the obtained nanoparticles were tested on both multidrug-resistant and -sensitive cells for their cytotoxicity in vitro and on tumor growth reduction in vivo. Intriguingly, compared to unconjugated AgNPs and to the commercial antitumor drug doxorubicin, TAT-AgNPs manifested a 24-fold higher anti-tumoral effect in vitro [33].

### 3.4. Intracellular Pathways Triggered by AgNPs

**Figure 3:**
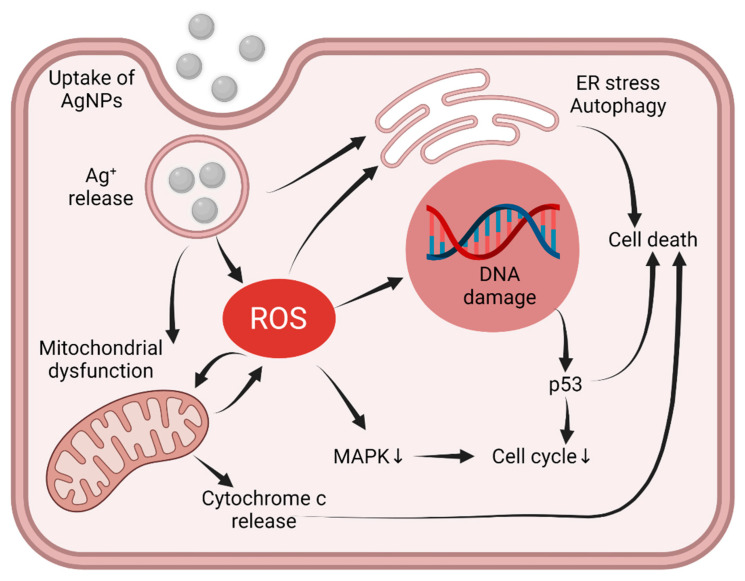
Following the uptake of AgNPs, Ag ions released from the nanoparticles contribute to the generation of reactive oxygen species. Ag ions directly or via oxidative stress cause mitochondrial dysfunction, ER stress, autophagy and DNA damage, leading to apoptosis. The figure was created with BioRender.com.

In order to understand the mechanisms of AgNP cytotoxicity, early studies aimed to reveal whether AgNPs themselves or the Ag ions released from AgNPs mediate the apoptotic events upon AgNP expositions [76,77,78]. Some studies reported that lysosomal entrapment is mandatory for enhanced Ag ion release. Since acidic pH facilitates the ionization of the AgNPs, this observation supports the hypothesis that Ag ions are the main drivers of the AgNP-triggered effects [79]. Additionally, it is acknowledged that silver ions drive the formation of ROS, which triggers massive oxidative stress, thereby activating the cellular pathways leading to cell death [65,80,81]. Importantly, scavenging AgNP-triggered ROS by antioxidants can hamper or prevent AgNP-induced cytotoxicity, illustrating the gravity of ROS involvement in AgNP toxicity [82,83]. Yet it seems that AgNP toxicity cannot be entirely mimicked only by inducing oxidative stress in the cells. It has been reported that cisplatin and AgNP treatments led to a comparable amount of ROS generation and similar anti-proliferative potency; however, in addition to apoptosis, cisplatin treatments provoked necrosis as well, while AgNP-treatments induced apoptosis only [84]. A potential explanation for this observation can be that while cisplatin treatment induces rapid cell death, AgNPs have a more prolonged effect on cell viability, the features of which can be ultimately exploited in therapy. Nevertheless, it is still a question of debate what sequence of events initiating from Ag ions results in the formation of ROS. Since mitochondria are the main sources of ROS, this organelle has been addressed as one of the main targets of intracellular Ag ions. Accordingly, mitochondrial dysfunctions, such as loss of mitochondrial membrane potential and mitochondrial structural disorganization, were reported to accompany the AgNP-induced stress [67,85,86,87]. These events can lead to cytochrome c release from the mitochondria into the cytoplasm and finally to apoptosis (Figure 3). However, ROS itself can also induce a similar molecular cascade [88]; therefore, further studies are mandatory to clarify whether mitochondrial stress is the cause or a consequence of Ag-induced ROS generation. 

Another issue is how intracellular AgNP accumulation and AgNP-induced oxidative stress advance to cancer cell death. AgNPs activate multiple signaling pathways, such as DNA damage-response and anti-proliferative signaling, by stimulating MAP kinases (Figure 3). Besides classical apoptotic pathways, sustained autophagy induction and ER-stress-related cell death were also described upon AgNP exposures [89,90] (Figure 3). In cancer cells, however, these major pathways are frequently altered; therefore, compared to normal cells, they might react differently to AgNP treatments [91,92,93]. Thus, understanding the response of cancer cells carrying various alterations in their major signaling pathways is mandatory. 

Among the various cellular elements, ROS target genomic DNA; therefore, the intrinsic DNA-damage response capacity of the treated cancer cells might be a critical determinant for efficient chemotherapy. Usually, cancer cells have an elevated tolerance toward DNA damage and a high DNA damage repair capacity, features that contribute to the evolution of chemo- and radiotherapy resistance as well [94]. Evidence exists that AgNPs can realize their anti-tumor activity via inducing DNA damage in primary tumor tissues [95] (Figure 3). It has been shown that cells lacking XPF—a factor involved in nucleotide excision repair (ner)—are more vulnerable to AgNPs than XPF expressing cells [96]. DNA damage culminates in the activation of the tumor suppressor P53; therefore, it is not surprising that the P53-dependent apoptotic pathway is activated upon AgNP treatment [97,98] and that silencing the P53 expression hampers the AgNP-induced apoptosis and DNA fragmentation in normal bronchial epithelial cells [99]. On the other hand, we found that AgNPs can induce apoptosis in P53-deficient cancer cells as well, demonstrating that P53 activation is rather a consequence of the ROS-induced DNA damage than the main driver of AgNP-triggered apoptosis in some cancer cells [67] (Figure 3). 

MAPK pathways are pleiotropic signaling routes regulating diverse cellular features, such as proliferation and survival, and their regulation is frequently affected in cancer cells. Typically, these pathways are induced by extracellular signals; however, ROS species have emerged as intracellular players stimulating MAPK pathways [100,101]. Thus, ROS generation links AgNP toxicity to MAP kinases. Castiglioni et al. have shown that ERK1/2 phosphorylation is necessary to achieve extensive AgNP-induced cytotoxicity in bladder carcinoma cells [102]; however, another study demonstrated that the anti-proliferative effects of AgNPs were coupled to ERK and Akt dephosphorylation [103]. Another element of this signaling pathway, the p38 MAP kinase was also implicated in AgNP-driven apoptosis, as AgNP exposures increased the phosphorylation status of p38, which then activated the apoptosis effector caspase-3 [104].

Dysregulation of autophagy was recently considered as a novel hallmark of cancer; thus, intercepting autophagic flux by AgNPs seems to be a viable strategy in modern cancer therapy [105]. Several reports suggest that AgNPs can modulate autophagy in both cancerous and non-cancerous cells [98,106]. We have also demonstrated that AgNPs of various sizes can trigger autophagy in breast cancer cells [107]. In fact, autophagy-induction seems to be one of the key features of AgNPs to execute their cytotoxic effects both in normal and tumor cells [98,108]. However, it is important to note that transformation-related dysregulation of the autophagy machinery can also hinder the cytotoxic effects of AgNPs by pushing consequential autophagy to a pro-survival strategy, which is a mechanism observed in several tumors to resist therapeutic regimens, such as radiation therapy, chemotherapy, and targeted therapies [109,110]. In osteosarcoma (HOS) and hepatocellular carcinoma (huh7) cells, biosynthesized protein-capped AgNPs restored autophagy as part of a pro-survival mechanism [111]. Consistent with this, AgNP treatment was shown to be cytoprotective in HeLa cells, but co-treatment with the autophagy inhibitor wortmannin changed the outcome drastically and turned it toward a heavily cytotoxic conclusion [112]. 

As ROS are potent inducers of autophagy, it seems feasible that the mediator of AgNP-induced autophagy would be oxidative stress [113]. AgNP-related ROS give rise to oxidatively modified proteins, which become labeled for degradation and eventually activate autophagic flux via redox signaling [114,115]. Additionally, AgNPs may lead to mitotoxicity-triggered mitophagy which is a form of selective autophagy [114,116,117]. In a recent study, AgNPs were shown to induce HiF-1α activation, thereby ultimately activating autophagy through the AMPK-mTOR pathway in PC-3 prostate cancer cells [89]. The mammalian Target Of Rapamycin (mTOR) is a downstream effector of PI3K/Akt and AMPK pathways, that is almost indispensable for the activation of autophagy. Nevertheless, in another study, sublethal doses of AgNPs were reported to induce ROS-independent autophagy via the same AMPK-mTOR signaling pathway, suggesting a ROS-independent but AgNP-triggered route of autophagy activation [118]. 

Perturbations that impede the protein folding machinery lead to ER stress and activate evolutionarily conserved unfolded protein response (UPR), which is mainly regulated by ER-resident chaperones, such as Bip and GRP94, and other stress sensors, such as IRE1α, PERK, and ATF6. UPR is primarily an adaptive response, which attempts to lower the number of proteins that enter the ER for folding, and it upregulates ER chaperones to assist in improving the folding of damaged proteins and relieve the burden of unfolded proteins on ER. Sustained ER stress drives UPR to the maladaptive response that activates programmed cell death mechanisms [119]. Although it has been shown that cancer cells manage ER stress better than non-cancerous cells, persistent ER stress triggers apoptosis in malignant cells as well; therefore, compounds leading to massive ER stress can have beneficial therapeutic effects [120].

AgNPs are capable of inducing ER stress based on the transcriptional and translational elevation of ER stress sensors PERK, IRE1α, and ATF6. Knocking down these stress sensors abolished AgNP-induced ER stress and thereby, apoptosis in human Chang liver cells and Chinese hamster lung fibroblasts, confirming the importance of ER stress-triggered cell death in AgNP toxicity [121]. As besides autophagy ROS are also key drivers of ER stress [122], it is not surprising that ER stress is one of the indispensable cellular consequences associated with AgNPs treatment [121]. Christen et al. showed that AgNPs induce ER stress in zebrafish embryos, which was again based on ROS generation [123]. Although mounting evidence supports that ROS mediate AgNP-induced ER stress, many studies have also reported ROS-independent ER stress elicited by AgNPs. A far more important aspect of AgNP-induced ER stress was shown in our recent study, where we demonstrated that ER stress is an underlying mechanism for attenuating multidrug resistance in breast cancer cells treated with AgNPs [107].

Based on the above-described findings, we can conclude that AgNP-induced oxidative stress triggers cellular damage, which affects almost every signaling axis of the cell. Therefore, it seems feasible that the signaling events observed upon AgNP treatments are solely due to the activation of oxidative stress (Figure 3). However, many studies point out that the molecular effects of AgNPs are far more complicated and cannot be precisely phenocopied by the induction of oxidative stress. Whether this feature can be exploited upon their therapeutic application needs to be carefully investigated in the future.

## 4. Applicability of AgNPs as Anti-Cancer Agents

### 4.1. Safety Issues: Toxicology on Healthy Tissues

The toxic aspects of AgNPs are emerging obstacles that need to be uncovered accurately in order to design safe and effective AgNP-based therapeutic tools. An important pharmacologic feature that dictates systemic AgNP toxicity is the biodistribution and tissue accumulation of AgNPs following administration. It has been revealed that nanoparticle biodistribution depends substantially on the size, shape, and surface charge of the nanomaterial, and therefore, organ-targeting can be fine-tuned and optimized by modifying these parameters [40]. Following intravenous administration, AgNPs are accumulated mostly in the liver and the spleen of the animals [124]. Liver-specific AgNP accumulation is not surprising, since intravenously injected pharmaceutics are inevitably transported into the liver through the portal vein and are subjected to liver filtering. However, since the spleen also gathers silver nanoparticles with high efficiency, it is reasonable that AgNPs are collected by the cells of the reticuloendothelial system (RES) and are, therefore, concentrated in RES organs. Thus, as expected, liver-resident macrophages—Kupffer cells—are the main scavengers of liver-accumulated nanoparticles [125]. Interestingly, dextran-coated AgNPs were able to evade RES cells and showed longer circulation time and lower spleen accumulation after systemic administration than non-coated counterparts [126]. Likewise, PEGylation has also been proposed as an alternative to increase plasma stability and reduce liver toxicity of silver quantum dots [127]. Kermanizadeh and coworkers demonstrated that one single bolus dose of AgNPs did not affect healthy liver functions in rats [128]; however, in another study, biochemical and histological evaluations revealed severe liver dysfunctions after an acute intravenous AgNP dose [129]. Therefore, how AgNP accumulation affects the function of these organs, in particular that of the liver, is not yet entirely clear. Importantly, AgNPs accumulated in high amounts in the cancerous tissue of solid tumor-bearing mice injected intravenously by radioactively labeled AgNPs. Apart from some nanoparticle accumulation in the liver and spleen of the animals, the high tumor-specific accumulation highlights that intravenously administered AgNPs can reach the cancerous tissue [130].

The nephrotoxicity of various AgNP-treated organisms was also examined. In fish, for example, it has been demonstrated that AgNPs could be trapped or filtered through the renal system [131]. However, a comparison of silver concentrations in the urine and feces of rats receiving AgNP administration revealed that the amount of silver eliminated through the urinary system is significantly less than those eliminated by fecal excretion, suggesting biliary secretion of AgNPs over renal filtering [132].

The possible harmful effects of AgNP treatments on brain functions were likewise investigated. AgNPs were detected in the brain of the experimental animals upon various administration routes, indicating that these nanoparticles may induce neurotoxicity [133,134]. Morphology and gene expression studies support this hypothesis, since AgNP administrations are often accompanied by neuronal cell shrinkage and altered gene expression profiles in the brain of the treated animals [135]. It has recently been reported that AgNPs can affect the integrity of the blood–brain barrier and can cross this barrier in vitro through transcytosis rather than via paracellular transport mechanisms [135,136]. Altered behavior of AgNP-treated rats seems to support this concept [137]; nevertheless, it has to be regarded with prudence, since AgNP treatments might influence the composition of the gut microbiota, which can ultimately lead to altered animal behavior as well [138].

### 4.2. Tailoring AgNP Surface Chemistry

Owing to their huge surface-size ratio, similarly to other nanomaterials, AgNPs are promising tools for targeted delivery. AgNPs possess the capacity to bind a wide range of organic chemicals and biomolecules mainly through chemisorption and physisorption. Commonly, AgNPs incubated in a solution of such bioactive compounds would carry these compounds in their corona. Chemisorption is a viable possibility, since the AgNP surface is highly reactive to form chemical bonds, such as ionic, covalent, and coordinate covalent bonds with various compounds. Due to strong Ag-S bonds, amino acids, peptides, and proteins are readily bound to the AgNPs surface. Similarly, N and O atoms are able to form bonds with silver ions; therefore, several organic compounds containing phenol, carbonyl, amide, hydroxyl, amino, and carboxyl groups have been reported to bind strongly to the surface of AgNPs. Physisorption, however, is mediated through weaker interactions, such as electrostatic interactions, hydrophobic entrapment, and van der Waals forces. Generally, these covalent and non-covalent binding properties of AgNPs are harnessed to achieve colloidal stability; however, in recent years, these features have been successfully exploited to formulate both active and passive anti-tumor drug delivery systems [139] using biocompatible AgNP synthesis methods, such as organic-water two-phase synthesis [140], micro-emulsion [141], radiolysis [142], and reduction in aqueous solution [104].

Recently, green synthesized AgNPs have been contemplated for clinical applications. Especially AgNPs synthesized using plant extracts as reducing agents gained prominent interest in nanomedicine, as plants are rich in phytochemicals, which can serve as coating agents during nanoparticle synthesis. Additionally, these AgNP synthesis approaches have the advantage of being eco-friendly, and the procedure is accessible, economical, simple to execute, and provides the possibility of large-scale production [143]. Khorrami et al. attempted to compare the tumor-selective cytotoxicity, the antioxidant, and antimicrobial properties of commercially available AgNPs, and of AgNPs generated by using the aqueous extract of walnut [144]. In this study, green synthesized AgNPs showed more prominent cancer-selective cytotoxicity and antioxidant properties than commercial counterparts, highlighting the advantages of green synthesized AgNPs over chemically generated ones. A further comprehensive review on green synthesized AgNPs and their medical implications are published by Ivanova et al. and by our group [12,143].

Although in vivo studies on bioconjugated and green-synthesized AgNPs are very limited, these AgNPs are commendable candidates in future nanomedicine, especially in cancer therapy. Major challenges in the progress of their employment are tumor cell selectivity, considerations on tumor heterogeneity and on the tumor stroma, as well as on critical parameters such as physiological barriers [145]. Tailoring the surface chemistry of AgNPs, more specifically, modifying their surface with various biologically active compounds for stabilization by capping, for functionalization aiming cancer cell targeting or drug delivery, requires more advanced studies to obtain therapeutically effective AgNP formulations. For a thorough view on the possibilities in surface functionalization of AgNPs readers are directed for reference [146]. 

### 4.3. Partners in Combination Therapy

To achieve maximal efficacy, in clinical practice, chemotherapeutics are often used in combinations. In such cases, drugs with synergistic effects are collected to target different vulnerable features of the cancer cells, thereby leading to an elevated eradication of the neoplastic mass. Even though AgNPs have a remarkable anti-cancer effect alone in vitro and in vivo, the most realistic therapeutic use of such nanoparticles might be their application as combinational partners with other anti-cancer agents upon chemotherapy or as auxiliary to other oncotherapeutic modalities. Accordingly, several studies focused on the cellular effects exhibited by AgNPs alone and jointly in combination with chemotherapeutic agents on cancer cells.

Using various ovarian cancer cells, Fahrenholtz and coworkers investigated the degree of mitochondrial dysfunction, apoptotic cell death, and autophagy induced by exposures to AgNPs or to AgNPs applied together with cisplatin. They found that AgNP treatments were effective on cell lines with higher intracellular basal ROS levels, but cells with a lower initial ROS level were not sensitive to AgNPs. Nevertheless, when AgNPs were administered in combination with cisplatin, they synergistically decreased the cell viability of the non-AgNP-sensitive ovarian cancer cells as well [60]. In another study, synergistic interactions were observed upon AgNP and salinomycin combinations. Salinomycin is an antimicrobial agent believed to be able to kill cancer stem cells; therefore, it represents a promising candidate for future chemotherapy [147]. AgNP treatment synergistically increased the salinomycin-induced mitochondrial dysfunction, autophagy, and apoptosis in A2780 human ovarian cancer cells. Both AgNP and salinomycin individual treatments induced ROS generation, the loss of mitochondrial membrane potential, and activated caspase-3 dependent apoptosis, which were significantly more pronounced upon combinational exposures [148]. Additionally, it has been shown that AgNPs and the topoisomerase I inhibitor camptothecin induce synergistically cervical cancer cell death. In this study, the combination of AgNPs and camptothecin increased ROS levels and induced LDH leakage, mitochondrial dysfunction, and apoptosis, while it reduced the level of antioxidants and decreased cell survival and cell proliferation [149].

As discussed above, eradication of multidrug-resistant (MDR) cancer cells is especially challenging for classical chemotherapy. AgNPs, however, may provide a solution to eliminate MDR cancer cells and overcome drug resistance. It has been established that AgNPs could affect many of the characteristic metabolic and cellular features of MDR cells, i.e., elevated tolerance to oxidative stress, increased apoptotic threshold, raised activation of DNA repair apparatus, and altered signal transduction pathways. Moreover, nanomaterials can hamper the increased efflux of anti-cancer agents through the inhibition of ATP-powered membrane pumps such as P-glycoprotein [107,150]. If drug efflux can be decreased as a result of AgNP action, chemotherapeutic drugs applied together with the nanomaterials would have a substantially bigger chance to exhibit their inherent anti-cancer mechanisms. Along this line, we investigated the efficiency of AgNP-drug combinations on Pgp-overexpressing drug-resistant Colo320 colon adenocarcinoma cells [150]. We tested functionally and structurally unrelated chemotherapeutic drugs (methotrexate, cisplatin, carmustine, bleomycin, vinblastine, and verapamil) as combinational partners of AgNP treatments. Based on cell viability data and calculated combinational indexes, synergistic interactions were verified for all tested chemotherapeutic drugs in combination with AgNPs, which suggested that AgNPs are able to enhance the efficacy of antineoplastic drugs upon treatments. Mechanistically, we demonstrated that modulated Pgp expression and/or efflux activity is the reason behind the observed synergistic interactions. In fact, ABC transport activity of multidrug-resistant cancer cells was decreased upon AgNP treatments, which in turn resulted in higher intracellular concentrations of chemotherapeutic agents leading to apoptosis in MDR cancer cells.

Since one of the cellular targets of AgNPs is the DNA and the chromatin structure, the combined action of AgNPs and chromatin structure modifying agents, such as histone-deacetylase (HDAC) inhibitors, was also examined [61]. Strong synergism was observed between the cytotoxicity of AgNPs and the HDAC inhibitor Trichostatin A (TSA) on HeLa cervical and on a further five cancer cell lines. Again, significantly higher oxidative stress and caspase-3 activity was observed upon the combinational treatment than following individual exposures to AgNP or TSA. Moreover, the genotoxic effect of AgNPs, indicated by the number of DNA double-strand breaks, was significantly more pronounced after AgNP-TSA combined exposures compared to the individual treatments. Thus, we concluded that HDAC inhibition leads to a relaxed chromatin structure, which renders the DNA more vulnerable to the damaging effects of Ag ions and the reactive oxygen species generated by AgNP treatment. Gurunathan and coworkers performed a similar study on A549 lung adenocarcinoma cells using the HDAC inhibitor MS-275 in combination with AgNPs. They found that the joint application of the nanoparticles and the HDAC inhibitor caused significantly increased ROS levels, and in agreement with this, notably decreased antioxidant levels compared to the individual treatments [151]. Moreover, AgNPs and MS-275 together triggered massive LDH leakage, induced TNF-alfa production, and significantly augmented apoptotic cell death than AgNPs or the HDAC inhibitor alone [151]. These findings indicated that the application of a mixture of metal nanoparticles and HDAC inhibitors is a viable combination that could be further exploited in other cancer treatment modalities as well.

The results of studies using combinational treatments of AgNPs and chemotherapeutic agents recommend the application of anti-cancer drugs simultaneously with AgNPs to increase the efficacy and to decrease the side effects of cancer therapy. Because of the synergism between AgNPs and several chemotherapeutic agents, lower concentrations of anti-cancer drugs might be sufficient to reach the same efficiency in cancer therapy; moreover, the damaging effects of anti-cancer agents could be decreased on non-tumoral cells and healthy tissues. 

### 4.4. Radio- and Photothermal Therapy

Apart from adding AgNPs simultaneously with classical chemotherapeutic drugs, another option of therapeutic AgNP combination can be realized together with radiation therapy. The idea to use AgNPs as radiosensitizers came along with the phenomenon that metals with high atomic numbers are capable of enhancing the effects of radiation. Mechanistically, this enhancement can be accomplished via physical, chemical, and biological dose enhancements, as was described in the case of gold nanoparticles [152]. Physical dose enhancement by metal nanoparticles is based on the high energy absorption and the induction of Auger cascade, Compton and photoelectric effects, and the release of secondary lower-energy electrons. On the other hand, the reactive electrons produced in this physical phase are transferred to oxygen molecules upon chemical dose enhancement, which contributes to the production of reactive oxygen species. As a consequence of injuries induced by ROS, if the biological system fails to repair the single and double-strand breaks induced in the DNA or the damages in the membrane structures, it leads to cell cycle arrest and finally to apoptotic or necrotic cell death [152].

Due to their plasmonic structure, AgNPs have the ability to scatter and absorb photons, which can be used in radiotherapy [153]. Comparing the efficiency of AgNPs with the radiosensitizing capacity of gold nanoparticles, it has been demonstrated that in glioma cells AgNPs increase the damaging effects of irradiation to a higher extent than gold particles [154]. Unlike for gold, the exact mechanism by which silver-based nanoparticles sensitize tissues for radiation therapy is still elusive; however, the radiosensitizing effect of AgNPs is probably dependent on the Ag ion release. It has been shown that smaller-sized AgNPs release more Ag ions and cause higher radioenhancement, while bigger AgNPs, with lower Ag ion releasing capabilities, were less effective [155].

In many tumors, the hypoxic conditions often suppress ROS generation, which hampers the efficacy of radiotherapy. Indeed, in such cases, AgNPs could yield an alternative solution by enhancing radiosensitivity. As a matter of fact, AgNPs caused the decrease of mitochondrial membrane potential and induced autophagy and apoptosis both in normoxic and in hypoxic irradiated glioma cells, indicating a potent radiosensitization exhibited by AgNPs [156].

The in vivo applicability of AgNPs as effective radiosensitizing agents has been demonstrated in a study, where AgNP treatment in combination with radiotherapy increased the life span of rats bearing gliomas without apparent toxicity. Mechanistically, AgNP exposure followed by irradiation inhibited cancer cell proliferation and in parallel induced apoptosis in glioma cells [157]. In another study, functionalized AgNPs were applied in combination with irradiation and inhibited effectively the progression of gliomas in mice. Here, AgNPs were functionalized with PEG and with a G-rich DNA aptamer As1411, which binds to nucleolin—a protein overexpressed on the surface of many cancer cells [70]. 

Besides radiosensitization, AgNP treatment is also capable of enhancing the effect of photo- and thermo-based therapies. For instance, it has been shown that AgNPs have excellent thermo-sensitizer activities on glioma cells. After AgNP exposure, the hyperthermia significantly decreased the colony-forming capabilities of glioma cells [158]. Furthermore, the combination of irradiation, magnetic-nanoparticle-mediated hyperthermia, and AgNPs was effectively utilized against cancer cells, since the administration of the three treatment modalities significantly decreased cancer cell survival compared to individual or dual treatments [159]. Finally, pectin-coated AgNPs increased the photo-induced cytotoxic effects of the organic photosensitizer riboflavin in cancer cells, as pectin-coated AgNPs enhanced the formation of ROS, generated by riboflavin after irradiation [160,161]. 

### 4.5. Therapeutic Strategies Implying Silver-Based Nanoparticles

Many promising in vivo research data support the potential applicability of AgNPs in cancer therapy, either as active component of complex nanosystems, or as combination partners of other therapeutic modalities (Table 2). However, their applicability as anti-cancer agents; thus, their clinical translation has still never been addressed. AgNPs are already commercialized to manage various clinical conditions, including the treatment of wounds and burns [162]. The utilization of AgNPs upon wound management has a long history, and fundamental research confirms that besides the antimicrobial effects, AgNPs can aid the tissue regeneration process via stimulating wound-resident fibroblasts, immune cells, and keratinocytes [163]. For such purposes, AgNPs are incorporated into textiles, creams, or can be integrated into other delivery matrices; thus, by topical application, they could locally exert their beneficial effects [164]. As a matter of fact, similar concepts have already been translated recently to cancer management using other materials, since it was recognized that post-operative cancer wound management can minimize the chance of metastasis and tumor relapse. Usually, following surgical resection of solid tumors, post-operative systemic chemotherapy is routinely applied to avoid tumor recurrence and suppress the dissemination of the remaining cancer cells. Therefore, to minimize systemic effects, novel formulations of clinically applied chemotherapy agents have been designed and developed, such as anti-cancer drug-releasing wafers, sprays, and foams, and utilized typically upon brain and breast cancer management, to locally treat the surgical lesion [165]. For example, temozolomide-releasing wafers could replace systemic chemotherapy upon post-operative glioblastoma care since they suppress tumor relapse more efficiently than systemic drug administration by inhibiting locally the persistent cancer cells [165]. Delivery matrices, gels, and hydrogels are often applied for post-operative, local cancer therapies, given that they are able to accurately follow the shape of the surgical wound. Using these delivery matrices AgNPs could be employed similarly for these clinical applications. Although AgNPs have not been tested in similar post-operative applications, they have already been incorporated successfully into hydrogels, creams, and other biocompatible polymers [166,167,168]. Hence, it seems that apart from the confirmed anti-cancer and anti-metastatic effects, AgNPs could be highly advantageous in this kind of surgical wound management; therefore, AgNPs should be tested in the future upon post-operative in situ cancer therapies.

Although surgical excision is the first-line therapy in skin cancer treatment, similarly to wound management, melanoma and non-melanoma cancers as well as pre-cancerous skin lesions are often treated in situ pre- or post-operatively. Considering the adverse effects of systemic chemotherapy, local treatment is generally more favorable. As such, topical creams, radiation, photodynamic therapy, and cryotherapy are frequently considered as alternative strategies for skin cancer management [169]. Notably, AgNPs are reported to be efficient against skin cancer cells in melanoma models [84,170]; therefore, we believe that AgNPs, as active components of textiles, creams, or gels, could be potent upon in situ skin cancer management as well.

## 5. Concluding Remarks—Future Perspectives

Based on numerous publications in recent years, it seems obvious that nanoparticle-based treatment of various tumors is regarded as a novel, attractive strategy to improve cancer therapy. These materials, such as AgNPs, can accumulate in the tumor tissues, interact with the different cancerous and stromal cells in this microenvironment, get internalized into the cells, where they activate a number of signaling pathways, and trigger mitochondrial dysfunction, oxidative stress, autophagy, ER stress, and various ways of cell death. Apart from a direct anti-cancer activity, AgNPs might serve as delivery platforms of various cytotoxic drugs or enhance the anti-cancer performance of combinational partners upon chemo- or radiotherapy. The plethora of such findings advocate that cancer therapy by such nanoparticles may soon turn fiction to reality. However, there is a lingering question: how to proceed? 

**Figure 4:**
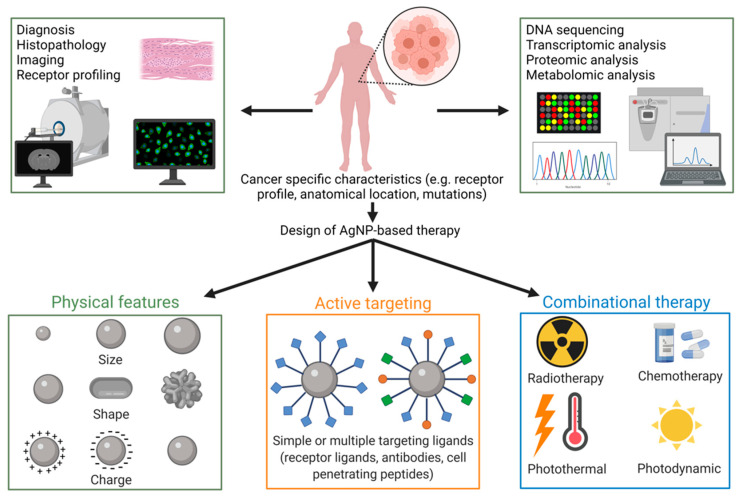
Merging modern diagnostic approaches provided by personalized cancer management and rational AgNP design can improve the anti-cancer efficiency of AgNPs. A detailed tumor phenotyping based on imaging and traditional histology techniques, together with high-throughput molecular characterization methods, can aid to select and design the ideal nanoparticle candidate with optimal size/shape, targeting the moiety and therapeutic combinational partner for each patient. The figure was created with Biorender.com.

Since cancer therapy by silver nanoparticles is a complex task, the first duty is to understand and characterize cancer. Tumors are highly heterogeneous; therefore, besides conventional histopathologic analysis and imaging techniques, receptor profiling and high-throughput personalized tumor analysis, such as DNA sequencing and transcriptomic, proteomic, and metabolomic analyses, are required to delineate mutations, signaling pathways, and cellular features that have been compromised upon tumor development and progression. Based on these data, personalized and patient-specifically tailored multifunctional silver nanoparticles should be produced. These tunable features include proper size/shape and surface charges ideal for an optimal pharmacokinetics, including tumor-specific AgNP accumulation. In addition, surface modifications for active cancer cell targeting, e.g., by conjugating AgNPs with receptors ligands, antibodies, and cell penetrating peptides, should also be taken into consideration for a customized nanoparticle design. Finally, the application of such sophisticated AgNPs is to be complemented with synergistically enhancing therapeutic approaches, e.g., chemo- and radiotherapy. However, prior to any of these utilizations, a detailed toxicology screening has to be carried out to secure biocompatibility and a safe and efficient AgNP-based oncotherapy (Figure 4). Despite the present challenges, there is no doubt that translation of metal nanoparticles to approved clinical treatment regimens is not far away.

## Figures and Tables

**Table 1:** Methods used for AgNP characterization.

Characterization Methods	Application	References
UV-Visible spectroscopy (UV-Vis)	Size, shape, stability and surface properties of nanoparticles, purity of sample	[13]
Scanning electron microscopy (SEM)	Size, shape, surface properties, purity of sample	[14]
Transmission electron microscopy (TEM)	Size distribution, shape, dispersity, purity of sample	[15]
Fourier transformed infrared spectroscopy (FT-IR)	Identification of surface residues, chemical species or functional groups	[16]
Powder X-ray diffraction (XRD)	Morphology, crystal structure, phase identification and crystallite size, purity of sample	[17]
Energy dispersive spectroscopy (EDS)	Structure and purity by determining the elemental composition	[18]
Atomic force microscopy (AFM)	Size, shape, surface properties, purity of sample	[19]
Dynamic light scattering (DLS)	Size distribution, average hydrodynamic diameter and stability	[20]
Zeta-potential measurement (ZP)	Stability and surface charge determination	[20,21]
Thermogravimetric analysis (TGA)	Chemical composition and the amount of coating on the surface of nanoparticles, thermal stability of nanoparticles	[22]
Inductively coupled plasma mass spectrometry (ICP-MS)	Surface chemical structure and chemical composition	[23]
Raman spectroscopy	Identification of surface residues, chemical species and functional groups	[24]
X-ray photoelectron spectroscopy (XPS)	Surface chemical composition, determination of chemical bonds	[25]

**Table 2:** Application of silver nanoparticles in in vivo cancer models.

Nanoparticle Applied	Feature	Model	Effect	Role of AgNPs	Ref.
AgNP-TAT	Cell penetrating peptide-functionalized NP	B16 melanoma xenograft	Reduced tumor growth	Ag as active compound	[33]
Ag/AuNP	Gold-silver alloy particles	Diethylnitrosamine-induced hepatocarcinogenesis	Reduced tumor growth	Ag as active compound	[171]
AgNP	PVP-coated particles	C6-glioma bearing rat	Increased life span, enhanced efficacy of radiation therapy	Ag as active compound	[156]
AgNP	PVP-coated particles	MDA-MB-231 TNBC xenograft in mice	Reduced tumor growth	Ag as active compound	[172]
Ag@AuNP	Au shell on AgNPs	PC-3 prostate carcinoma xengraft in mice	Increased tumor growth inhibition by photothermal therapy	Ag as active compound	[173]
Ag/Ali@PNPs–Cltx	Silver/alisertib@polymeric nanoparticles conjugated with chlorotoxin	U87MG glioblastoma Xenograft in mice	Decreased tumor size	AgNP for delivery	[49]
QagNP	Quinacrine-based hybrid silver NP	SCC-9 head and neck cancer cells xenograft in mice	Decreased tumor size	AgNP for delivery	[174]
Tat-FeAgNP-Dox	Dextrin-coated silver nanoparticles attached with iron oxide nanoparticles, cell penetrating peptide and loaded with doxorubicin	MCF-7 xenograft in mice	Reduced tumor growth	AgNP for delivery	[175]
rTL/ABZ@BSA/Ag NP	Albendazole encapsulated in albumin-coated AgNPs and modified with cell penetrating peptide	Xenograft of drug resistant A549/T cells, and metastasis to lung in mice	Reduced tumor growth and metastasis	AgNP for delivery	[176]
AsNP	Aptamer As1411-functionalized AgNP	C6-glioma bearing mice	Increased efficacy of radiation therapy and life span	Ag as active compound	[70]
Ag@TiO_2_NP	AgNPs in a TiO_2_ shell layer	B16-F10 mleanoma cell xenograft in mice	Inhibit tumor growth as a high-performance photothermal therapy agent	Ag as active compound	[170]
AgNP	PVP-coated particles	B16-F10 melanoma cell xenograft in mice	Reduced tumor growth and increased survival	Ag as active compound	[84]
pGAgNPs	PEGylated, graphene-decorated silver nanoprisms	HCT116 colorectal cancer cell xenograft-bearing mice	Decreased tumour growth and increased life span by enhancing radiotherapy	Ag as active compound	[177]
AgNP-MSA	Mouse serum albumin-coated AgNPs	3-methylcholanthrene and 12-O-tetradecanoyl-phorbol-13-acetate-induced mice fibrosarcoma	Reduced tumor growth and decreased incidence	Ag as active compound	[178]
CNT/AgNPs	Carbon nanotube-decorated AgNPs	B16-F10 melanoma cell xenograft in mice	Decreased tumor size as a photothermal therapy agent	Ag as active compound	[179]
Au@Ag	Au core Ag shell nanoparticles	4T1 mice tumor metastasis model	Inhibition of lung metastasis	Ag as active compound	[50]
